# Robust estimation of the time-dependent reproduction number in the presence of weekend reporting effects

**DOI:** 10.1186/s44263-026-00280-z

**Published:** 2026-05-21

**Authors:** Isaac Ogi-Gittins, Nicholas Steyn, Alexander R. Kaye, Edward M. Hill, Robin N. Thompson

**Affiliations:** 1 Mathematics Institute, University of Warwick, Coventry, UK; 2 Zeeman Institute for Systems Biology and Infectious Disease Epidemiology Research (SBIDER), University of Warwick, Coventry, UK; 3 Department of Statistics, University of Oxford, Oxford, UK; 4 Civic Health Innovation Labs, University of Liverpool, Liverpool, UK; 5 Department of Public Health, Policy and Systems, Institute of Population Health, University of Liverpool, Liverpool, UK; 6 National Institute for Health and Care Research (NIHR) Health Protection Research Unit in Emerging and Zoonotic Infections, University of Liverpool, Liverpool, UK; 7 The Pandemic Institute, Liverpool, UK; 8 Mathematical Institute, University of Oxford, Oxford, UK

**Keywords:** Mathematical modelling, Infectious disease epidemiology, Time-dependent reproduction number, Parameter inference, Day-of-the-week effect, Weekend reporting effect

## Abstract

**Background:**

During infectious disease outbreaks, changes in pathogen transmissibility are assessed through real-time inference of metrics such as the time-dependent reproduction number (*R*_*t*_), which can be estimated from disease incidence data. However, such data are often subject to a “day-of-the-week effect” (DOWE), whereby the number of cases on certain days of the week is liable to being under-reported (due to administrative delays) or over-reported (as public health authorities “catch-up” on reporting delayed cases). For example, cases occurring at weekends may only be reported during the following week, leading to under-reporting at weekends and over-reporting on weekdays (a weekend reporting effect; WRE).

**Methods:**

We analyse simulated datasets, as well as case reports from San Francisco recorded during the 1918 influenza pandemic. We investigate the impacts of WREs on *R*_*t*_ estimates obtained using two approaches: i) the Cori method (a frequently applied method for estimating *R*_*t*_ from daily disease incidence time series data); ii) an alternative method, involving aggregating the daily incidence data into weekly values to remove the WRE and applying a previous method (the OG1 method) for inferring *R*_*t*_ from weekly data.

**Results:**

Our analyses indicate that *R*_*t*_ estimates obtained from standard approaches such as the Cori method can be affected negatively by WREs. In contrast, since weekly aggregation of daily data can eliminate WREs, the alternative approach generates robust *R*_*t*_ estimates in the presence of WREs. When aggregating the daily data into weekly values, some information is lost. However, in many scenarios, the negative impact of data aggregation on *R*_*t*_ inference is outweighed by the benefit of then using data that are not corrupted by a WRE.

**Conclusions:**

Our research highlights the importance of accounting for DOWEs, such as WREs, when estimating *R*_*t*_ during infectious disease outbreaks.

## Background

Tracking pathogen transmission during an infectious disease outbreak, for example through real-time inference of the time-dependent reproduction number (*R*_*t*_), is useful to guide public health policy decisions [1–4]. In addition, estimates of *R*_*t*_ can be used to inform the public about changes in transmission [5], potentially improving adherence to public health measures [6, 7]. For example, during the COVID-19 pandemic, *R*_*t*_ estimates were used by policy advisors to explain the current state of the outbreak to the public, and were published on public health dashboards in countries worldwide.

While *R*_*t*_ is a valuable metric for quantifying transmission, *R*_*t*_ estimates are only practically useful if they are accurate. If ${R}_{t} > 1$, its precise value determines the proportion of transmissions that must be prevented to bring the outbreak into decline. Similarly, if ${R}_{t} < 1$, its value determines the additional transmission that could occur without a resurgence in cases. Consequently, public health measures based on inaccurate *R*_*t*_ estimates could lead to unnecessarily strict interventions being introduced (when ${R}_{t} > 1$) or interventions being relaxed inappropriately so that case numbers rise again (when ${R}_{t} < 1$). In addition, short-term projections using incorrect *R*_*t*_ values could lead to misinformed healthcare capacity planning, including suboptimal healthcare service staffing, hospital or intensive care unit bed provision and contact tracing resourcing. The efficacy of implemented public health measures can sometimes be inferred based on changes in estimated *R*_*t*_ values [8–10]. Biased estimates can therefore lead to incorrect conclusions about which interventions are most effective. When inaccuracies arise from systematic reporting artefacts, such as “day-of-the-week effects” (DOWEs, which we explain in more detail below), inferred *R*_*t*_ values may reflect administrative processes rather than epidemiological changes. Ensuring that *R*_*t*_ estimates are robust to reporting biases is therefore essential for reliable situational awareness during outbreaks.

Mathematical modellers have developed a range of methods for inferring *R*_*t*_ [11]. A commonly used approach is the Cori method [1, 12], which allows *R*_*t*_ estimates to be obtained from two inputs: (i) case data (a disease incidence time series); (ii) the serial interval distribution (a probability distribution characterising the period between symptom onset times in infector-infectee transmission pairs [13]). We note that, if the case data comprise infection times rather than symptom onset times, then the Cori method may still be applied but the serial interval distribution should be replaced by the generation time distribution (which characterises the period between infection times in infector-infectee transmission pairs [14–16]).

While the Cori method is simple to apply, it relies on its inputs being accurate. For example, as noted by Gostic *et al*. [17], methods for inferring *R*_*t*_ can be sensitive to misspecification of the generation time. Similarly, it might be expected that inaccurate *R*_*t*_ estimates may be obtained from biased or otherwise misreported disease incidence time series datasets. A key potential source of bias in disease incidence time series data is a DOWE, whereby incidence data display variations not only because of the progression of the epidemic but also due to the day of the week [18]. A common DOWE is the “weekend reporting effect” (WRE), which is widely observed in epidemiological data, with cases occurring at weekends often being subject to delayed reporting. This can arise due to limited access to healthcare services at weekends. For example, in the United Kingdom, General Practitioners were not required to offer services on Saturdays until 2022, and most remain closed on Sundays. Similarly, in the Netherlands, health reporting guidelines differ at weekends compared to weekdays; General Practitioners must report laboratory-confirmed cases of high risk diseases to health authorities within one day, however this requirement is relaxed to three days around weekends [19].

WREs have been observed in numerous epidemiological datasets. For example, Wei *et al*. [20] analysed data describing cases of hand, foot and mouth disease and epidemic parotitis (mumps) in Hanzhong, China, from 2014 to 2017. Those authors identified that cases were least likely to be reported on weekend days and most likely to be reported on Mondays, reflecting delays in reporting over weekends. Similarly, Simpson *et al*. [21] analysed COVID-19 data spanning March 2020 to November 2021 from Massachusetts, United States of America, and found that test results were reported at approximately twice the rate on weekdays compared with weekends. Amirov *et al*. [22] analysed the reporting of enteric and respiratory outbreaks in healthcare settings from 2006 to 2010, finding a substantially lower probability of outbreaks being reported at weekends compared to other days of the week. While some of the healthcare settings in question, such as long-term care facilities, remained open at weekends, the authors noted that reporting and validation of outbreaks often required infection control personnel to be present, which typically did not happen at weekends.

In this article, we investigate the potential impacts of WREs on estimates of pathogen transmissibility during infectious disease outbreaks by comparing results from two methods for estimating *R*_*t*_. Under the first approach, we apply the widely used Cori method to daily disease incidence time series data. Under the second approach, we first aggregate the daily disease incidence data into weekly values, and then apply a simulation-based method that has previously been proposed for estimating *R*_*t*_ from weekly aggregated disease incidence time series data [23]. We refer to the second approach as the OG1 method, following the use of this terminology previously [24], although the deliberate weekly aggregation of daily disease incidence data is specific to the current manuscript.

We conduct a range of analyses to investigate the impact of WREs on estimates of *R*_*t*_. We first analyse simulated datasets to compare *R*_*t*_ estimates obtained using the Cori method applied to daily disease incidence data against *R*_*t*_ estimates obtained from the OG1 method (following weekly aggregation of the disease incidence data, as described above). We then explore how the magnitude of the WRE and the underlying value of *R*_*t*_ influence the performance of these approaches. Finally, we extend our analyses to a realistic epidemiological setting by considering data derived from weekly case reports from San Francisco recorded during the 1918 influenza pandemic, reconstructing daily incidence and examining the effects of different levels of WRE on *R*_*t*_ estimates obtained from the Cori and OG1 methods.

## Methods

Here, we outline the methods used in this study. We first describe our implementation of the Cori method and the OG1 method. Although we assume that daily disease incidence data are available, the goal of both methods is to infer the value of *R*_*t*_ each week (under the assumption that *R*_*t*_ is constant within weeks). We then describe the serial interval distribution used in all our analyses. Finally, we describe the simulated and real-world datasets that we use to explore the effects of WREs on the accuracy of *R*_*t*_ estimates.

### Cori method

We applied the Cori method to daily disease incidence time series data (denoted ${I}_{1}^{{\rm{daily}}}$, ${I}_{2}^{{\rm{daily}}}$, ${I}_{3}^{{\rm{daily}}}$ and so on) to infer the value of *R*_*t*_ each week (for $t\ge 2$ weeks). In other words, as noted above we estimated weekly values of *R*_*t*_ from the daily data under the assumption that *R*_*t*_ is constant during each week. The incidence data in week $t=1$ are ${\{{I}_{s}^{{\rm{daily}}}\}}_{s=1}^{7}$, the incidence data in week $t=2$ are ${\{{I}_{s}^{{\rm{daily}}}\}}_{s=8}^{14}$, and so on.

In the transmission model underlying the Cori method, it is assumed that the number of cases each day is drawn from a Poisson distribution. Denoting the number of cases on day *i* of week *t* by ${I}_{7\left(t-1\right)+i}^{{\rm{daily}}}$ (for $i=\mathrm{1,2},\ldots ,7$), the mean of the corresponding Poisson distribution is

1 $${\mathbb{E}}\left({I}_{7\left(t-1\right)+i}^{{\rm{daily}}}\right)={R}_{t}\mathop{\sum }\limits_{s=1}^{7\left(t-1\right)+i-1}{I}_{7\left(t-1\right)+i-s}^{{\rm{daily}}}{w}_{s},$$

where *w*_*s*_ is the probability that the serial interval takes the value *s* days. To infer *R*_*t*_ from the daily incidence data observed up to and including week *t*, we assumed that the prior for *R*_*t*_ is a gamma distribution with shape parameter *α* and rate parameter *β*. In our analyses, as in previous publications [12, 23], we set $\alpha =1$ and $\beta =0.2$, resulting in a relatively uninformative prior for *R*_*t*_.

Following previous publications in which the Cori method has been used (see e.g. [23]), and assuming an estimation window of duration one week (so that the posterior distribution for *R*_*t*_ is based on cases observed in the period from day $7\left(t-1\right)+1$ to 7*t*), then the posterior distribution for *R*_*t*_ is also a gamma distribution, with shape parameter $\alpha +\mathop{\sum }\limits_{s=1}^{7}{I}_{7\left(t-1\right)+s}^{{\rm{daily}}}$ and rate parameter $\beta +\mathop{\sum }\limits_{k=1}^{7}\mathop{\sum }\limits_{s=1}^{7\left(t-1\right)+k-1}{I}_{7\left(t-1\right)+k-s}^{{\rm{daily}}}{w}_{s}$.

We note that, in the original publications reporting the Cori method [1, 12], a sliding estimation window was used, enabling different *R*_*t*_ estimates to be generated every day (based, for example, on the cases observed on that day and each day in the preceding week). Since our goal was to estimate *R*_*t*_ each week, we did not “slide” the estimation window and instead inferred *R*_*t*_ in any week based on the cases observed in that week, as described above.

### OG1 method

To infer *R*_*t*_ using the OG1 method (for $t\ge 2$ weeks), we first aggregated the daily disease incidence into weekly values (denoted ${I}_{1}$, ${I}_{2}$, and so on). In other words, we set ${I}_{t}=\mathop{\sum }\limits_{i=1}^{7}{I}_{7\left(t-1\right)+i}^{{\rm{daily}}}$ for $t=\mathrm{1,2,3,}\ldots$. We note that, when aggregating daily data into weekly values, there are seven possible choices for how weeks are defined; weeks could be defined as running from Monday to Sunday, or from Tuesday to Monday, or from Wednesday to Tuesday, and so on. In our analyses, we assumed that the WRE involves some cases occurring on Saturdays and Sundays being reported on the subsequent Monday. If weeks are assumed to run from Monday to Sunday, this leads to some weekend cases being recorded in incorrect weeks. As a result, we instead chose to aggregate the daily data into weeks running from Wednesday to Tuesday (although of course other appropriate choices could have been made). Consequently, under this approach, if an outbreak started on a Monday, then ${I}_{1}^{{\rm{daily}}}$ would correspond to the previous Wednesday, with ${I}_{i}^{{\rm{daily}}}=0$ for $i=\mathrm{1,2},\ldots ,5$ and the index cases being assigned to ${I}_{6}^{{\rm{daily}}}$.

Following [23], we then estimated *R*_*t*_ (for $t=\mathrm{2,3,4},\ldots$) from the weekly aggregated data via repeated simulation of a renewal equation model with a daily timestep (i.e., where the number of cases each day is drawn from a Poisson distribution with mean given by Eq. (1)). To infer *R*_2_, we performed the following three steps repeatedly: (i) we assigned the ${I}_{1}$ cases in week one to days within that week (we sampled the day of each case uniformly between the known first day of the outbreak and the final day in week one); (ii) we sampled a value of *R*_2_ from the prior (i.e., from a gamma distribution with shape parameter *α* and rate parameter *β*); (iii) we simulated the renewal equation model with a daily timestep each day in week two (using the sampled daily incidence data in week one from step i and the sampled *R*_2_ value from step ii). Steps i-iii were repeated until $M=\mathrm{1,000}$ simulations had been generated in which the simulated number of cases in week two matched the number of cases in week two in the dataset (i.e., ${I}_{2}$). The matching simulations were stored. The mean estimate of *R*_*2*_ and the corresponding 95% credible interval were then calculated from the values of *R*_2_ used in the matching simulations.

*R*_*t*_ inference for $t\ge 3$ weeks followed similarly. Specifically, for each *t* in turn, we performed the following three steps repeatedly: (i) we sampled daily incidence up to (and including) week $t-1$ from the matching sets obtained when estimating ${R}_{t-1}$; (ii) we sampled a value of *R*_*t*_ from the prior; (iii) we simulated the renewal equation model with a daily timestep each day in week *t* (using the simulated incidence prior to week *t* sampled in step i and the *R*_*t*_ value sampled in step ii. Steps i-iii were again repeated until $M=\mathrm{1,000}$ simulations had been generated in which the simulated number of cases in week *t* matched the number of cases in week *t* in the dataset, and the matching simulations were again stored. The mean estimate of *R*_*t*_ and the corresponding 95% credible interval were then calculated from the values of *R*_*t*_ used in the matching simulations.

### Serial interval

From household data, Cauchemez *et al*. [25] obtained a serial interval estimate for pandemic influenza with mean 2.6 days and standard deviation 1.3 days. In all our analyses, we therefore used a discrete serial interval distribution ${\{{w}_{s}\}}_{s=1}^{100}$ that approximates a (continuous) gamma distribution with mean 2.6 days and standard deviation 1.3 days. As described in our earlier publications [23, 26], to discretise the continuous serial interval distribution we set

$${w}_{s}=\underset{s-1}{\overset{s+1}{\int }}g\left(u\right)\left(1-|u-s|\right){du},$$

for $s=\mathrm{2,3},\ldots ,100$, in which $g\left(u\right)$ is the probability density at value *u* of a gamma distribution with mean 2.6 days and standard deviation 1.3 days. We then chose ${w}_{1}$ so that ${\{{w}_{s}\}}_{s=1}^{100}$ is a valid probability distribution (i.e., $\mathop{\sum }\limits_{s=1}^{100}{w}_{s}=1$).

### Simulated datasets

We tested the performance of the Cori method (using daily disease incidence data) and the OG1 method (after aggregating the daily disease incidence data into weekly values) on a range of simulated datasets.

To generate a simulated dataset, we first simulated the true number of cases each day, neglecting the WRE. To do this, we simulated a renewal equation model in which the number of cases each day is drawn from a Poisson distribution with mean given by Eq. (1). Outbreaks were assumed to start with the index cases occurring on a Monday. As described in the “OG1 method” subsection of the Methods, when we applied the OG1 method we aggregated the daily disease incidence data into weekly values with weeks running from Wednesday to Tuesday. Consequently, in each simulated dataset, ${I}_{i}^{{\rm{daily}}}=0$ for $i=\mathrm{1,2},\ldots ,5$ with the index cases being assigned to ${I}_{6}^{{\rm{daily}}}$.

**Fig. 1:**
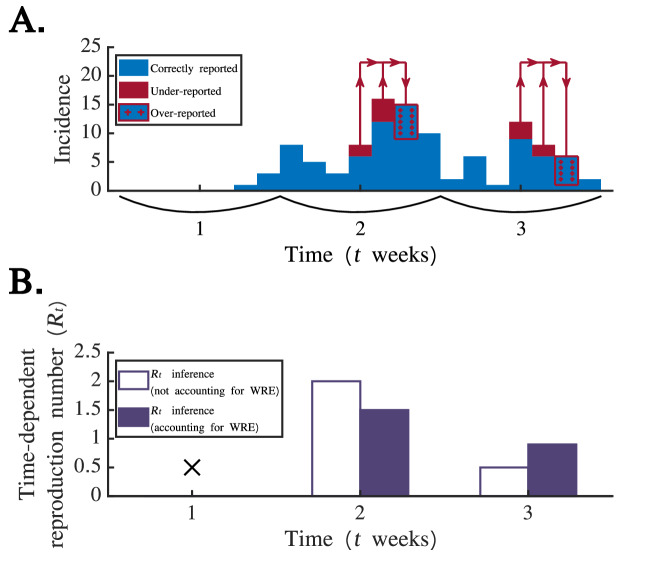
Schematic illustrating the assumed impact of a weekend reporting effect (WRE) on daily disease incidence time series data and the potential consequences for *R*_*t*_ estimates obtained using standard inference methods. **A**. Disease incidence time series data for ${p}_{W}=0.75$. In the dataset, 100% of cases reporting symptoms on weekdays and (on average) 75% of cases reporting symptoms at weekends are reported on the correct days (blue). The remaining (on average) 25% of weekend cases (red) are subject to delayed reporting, only being reported on the subsequent Monday (blue with red crosses). This leads to incorrect daily disease incidence data being used as inputs to the Cori method. In contrast, when the OG1 method is applied, the daily data are aggregated such that each weekend and subsequent Monday fall within the same week (curved lines under the x-axis), leading to correct weekly disease incidence data. **B**. Example inference of *R*_*t*_ for the dataset in panel A using the Cori method, either using the incorrect daily disease incidence time series that have been subjected to the WRE (as would be observed in practice; white) or using the correct daily disease incidence time series from before the application of the WRE (as would not be observed in practice; dark blue). As illustrated here, WREs can lead to incorrect estimates of *R*_*t*_ inferred directly from the observed daily data

The final version of each simulated dataset was obtained by applying a WRE to the simulated time series of true cases. The extent of the WRE was characterised by *p*_*W*_, which represents the probability that a case that occurs at the weekend is reported on the correct day. Consequently, for each true case that occurred on a weekend day, we sampled whether it was reported on that day from a Bernoulli distribution with probability *p*_*W*_. If it was not reported on that day, it was recorded in the simulated dataset on the subsequent Monday (see Fig. 1A for a schematic illustrating this procedure).

We generated three sets of simulated datasets. In the first set, we assumed that there were 10 index cases, that ${R}_{t}=1.5$ in weeks $t=\mathrm{1,2},\ldots ,7$ (growth phase of the outbreak) and ${R}_{t}=0.75$ in weeks $t=\mathrm{8,9},\ldots ,12$ (decline phase of the outbreak). We generated 1,000 “true” daily disease incidence time series in this fashion. For each of these time series, we then created 101 separate simulated datasets, with each dataset corresponding to a different WRE (for a single value of ${p}_{W}=0,\,0.01,\,0.02,\,\ldots ,\,1$). In total, this procedure generated 101,000 simulated datasets.

The second and third sets of simulated datasets were generated to test the performance of the Cori and OG1 methods on synthetic datasets produced using a range of *R*_*t*_ values. In the second set of simulated datasets, we simulated only the growth phase of the outbreak (i.e., weeks $t=\mathrm{1,2},\ldots ,7$). We initiated each of 1,000 simulations with 30 index cases, and sampled the value of *R*_*t*_ from a uniform $U(1,\,1.75)$ distribution. For each simulated “true” disease incidence time series, we simulated three datasets with weekend effects corresponding to ${p}_{W}=0.25,\,0.5$ and 0.75. This led to 3,000 simulated datasets in total (each consisting only of the growth phase of the outbreak).

In the third set of simulated datasets, we simulated only the decline phase of the outbreak (i.e., weeks $t=\mathrm{8,9},\ldots ,12$). In each simulation, we used a disease incidence time series for the growth phase of the outbreak from the first set of simulated datasets (in which ${R}_{t}=1.5$ in the growth phase); we used a different time series from the first set of simulated datasets in each simulation. We then sampled the value of *R*_*t*_ in the decline phase of each outbreak from a uniform $U(0.55,\,1)$ distribution and simulated from $t=8$ weeks onwards. Again, for each simulated “true” disease incidence time series, we simulated three datasets with weekend effects corresponding to ${p}_{W}=0.25,\,0.5$ and 0.75, generating 3,000 simulated datasets in total (and we analysed the decline phases of each of these outbreaks).

### Real-world dataset: Influenza in San Francisco, 1918

To investigate how the two methods for estimating *R*_*t*_ perform for realistic disease incidence time series data, we considered weekly influenza case reports from San Francisco for the nine-week period from 23 September to 24 November 1918 [27]. During this period, 28,310 cases were reported.

Since we aimed to test how the inference methods perform for daily data that are subject to different WREs, we first reconstructed the daily data underlying the weekly case reports. To do this, we fitted a deterministic Susceptible-Exposed-Infectious-Removed model [28] to the weekly data, inferring the daily case numbers (see Supplementary material 1: Supplementary text and Supplementary material 1: Fig. S1). We then generated three datasets by implementing WREs corresponding to ${p}_{W}=0.25$, 0.5 and 0.75. In each of these scenarios, we again implemented WREs by sampling whether each weekend case was reported on the correct day from a Bernoulli distribution with probability *p*_*W*_. Again, if a weekend case was not reported on the correct day, then it was assumed to be reported on the subsequent Monday.

## Results

### Simulated datasets

**Fig. 2:**
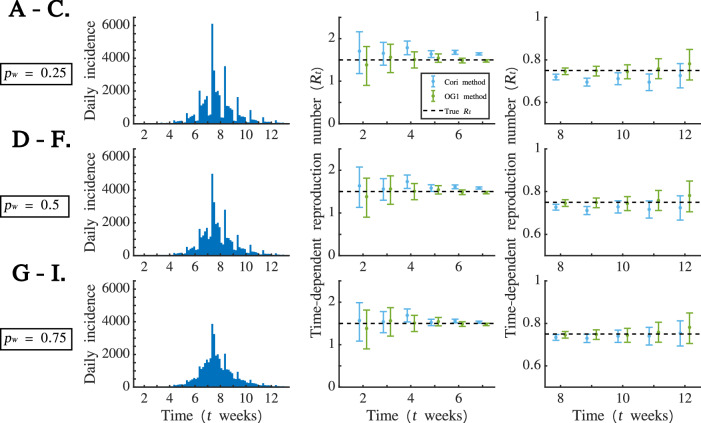
Using three simulated datasets to investigate the effects of WREs on *R*_*t*_ estimates from the Cori and OG1 methods. **A**. Randomly chosen disease incidence time series dataset in which ${p}_{W}=0.25$, ${R}_{t}=1.5$ in the outbreak growth phase and ${R}_{t}=0.75$ in the outbreak decline phase. **B**. Estimates of *R*_*t*_ obtained from the Cori (blue) and OG1 (green) methods in the growth phase of the outbreak shown in panel A; dots represent mean estimates and error bars represent 95% credible intervals. The true underlying value of *R*_*t*_ is also shown (black dashed). **C**. Estimates of *R*_*t*_ obtained from the Cori (blue) and OG1 (green) methods in the decline phase of the outbreak shown in panel A; dots represent mean estimates and error bars represent 95% credible intervals. The true underlying value of *R*_*t*_ is also shown (black dashed). **D**-**F**. Analogous to **A**-**C**, but for the simulated dataset corresponding to panel A for which ${p}_{W}=0.5$. **G**-**I**. Analogous to **A**-**C**, but for the simulated dataset corresponding to panel A for which ${p}_{W}=0.75.$

As an initial investigation into the effect of WREs on *R*_*t*_ estimates obtained from the Cori and OG1 methods, we first randomly chose one of the simulated disease incidence time series generated with ${R}_{t}=1.5$ in the growth phase of the outbreak and ${R}_{t}=0.75$ in the decline phase of the outbreak (i.e., one simulation from the first set of simulated datasets described in the “Simulated datasets” subsection of the Methods). For the chosen simulation, we analysed the corresponding simulated datasets in which ${p}_{W}=0.25$ (Fig. 2A), ${p}_{W}=0.5$ (Fig. 2D) and ${p}_{W}=0.75$ (Fig. 2G). For each of these, we estimated *R*_*t*_ using the Cori and OG1 methods; estimates from the outbreak growth phase are shown in Fig. 2B (for ${p}_{W}=0.25$), Fig. 2E (for ${p}_{W}=0.5$) and Fig. 2H (for ${p}_{W}=0.75$), and estimates from the outbreak decline phase are shown in Fig. 2C (for ${p}_{W}=0.25$), Fig. 2F (for ${p}_{W}=0.5$) and Fig. 2I (for ${p}_{W}=0.75$).

We found that the OG1 method generally leads to more robust estimates of *R*_*t*_ than the Cori method. For example, for ${p}_{W}=0.5$, not only were the mean *R*_*t*_ estimates closer to the true value of *R*_*t*_ under the OG1 method compared to under the Cori method for nine out of 11 inference points (Fig. 2E and F), but the true value of *R*_*t*_ also lay within all 11 credible intervals when the OG1 method was applied (compared to only lying in five out of 11 credible intervals when the Cori method was applied).

We also found that the Cori method generated overestimates of *R*_*t*_ when ${R}_{t} > 1$ (every mean estimate of *R*_*t*_ was greater than the true value) and underestimates of *R*_*t*_ when ${R}_{t} < 1$ (every mean estimate of *R*_*t*_ was less than the true value).

**Fig. 3:**
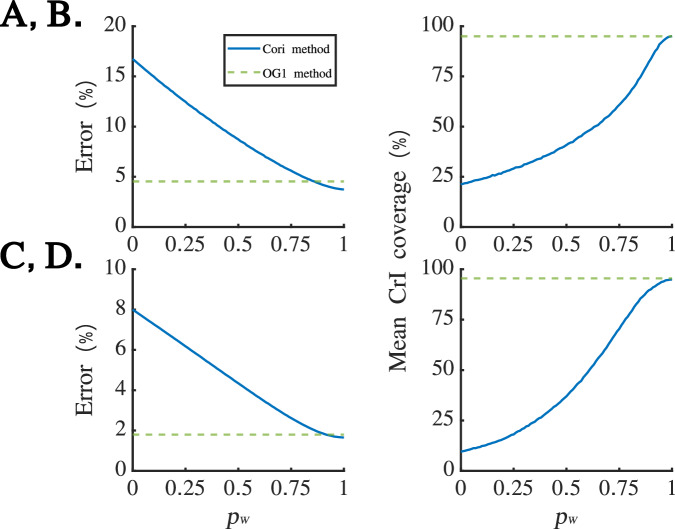
Using 101,000 simulated datasets with different WREs to investigate the effect of *p*_*W*_ on the accuracy and coverage of *R*_*t*_ estimates from the Cori and OG1 methods. **A**. Mean error (compared to the true underlying value of ${R}_{t}=1.5$) in mean estimates of *R*_*t*_ obtained in the outbreak growth phase from the Cori method (blue) and OG1 method (green dashed), across 101,000 simulated datasets (1,000 datasets for each considered value of *p*_*W*_). **B**. Credible interval coverage (percentage of estimates for which the true value of ${R}_{t}=1.5$ lies within the 95% credible interval of the posterior for *R*_*t*_) for estimates obtained in the outbreak growth phase from the Cori method (blue) and OG1 method (green dashed), across 101,000 simulated datasets. **C**-**D**. Identical to **A**-**B**, but for the outbreak decline phase (in which ${R}_{t}=0.75$)

We then repeated this analysis for all 101,000 simulated datasets for which ${R}_{t}=1.5$ in the outbreak growth phase and ${R}_{t}=0.75$ in the outbreak decline phase (i.e., all simulated outbreaks in the first set of simulated datasets described in the “Simulated datasets” subsection of the Methods). For each dataset, in each week we calculated the absolute value of the percentage error in the mean *R*_*t*_ estimate (compared to the true value of *R*_*t*_ in that week). Separately for each value of *p*_*W*_, and separately for the outbreak growth and decline phases, we then calculated the mean of this quantity across all datasets and weeks. Results are shown in Fig. 3A for the outbreak growth phase and Fig. 3C for the outbreak decline phase. Since the OG1 method involves first aggregating the daily disease incidence into weekly values, thereby eliminating the WRE, the error in mean *R*_*t*_ estimates from the OG1 method is independent of *p*_*W*_ (green dashed lines in Fig. 3A and C). Unsurprisingly, in general we found that the Cori method performed well for values of *p*_*W*_ close to one (i.e., when there was little or no WRE) but relatively poorly when *p*_*W*_ was substantially less than one. Interestingly, when ${p}_{W}=1$, mean estimates from the OG1 method were only slightly less accurate than from the Cori method, suggesting that aggregating the daily disease incidence data into weekly values had a relatively small effect on the accuracy of *R*_*t*_ estimates.

As well as assessing the accuracy of the mean *R*_*t*_ estimates, we also considered the credible interval coverage (i.e. the percentage of estimates for which the 95% credible interval contained the true *R*_*t*_ value) in both the growth (Fig. 3B) and decline (Fig. 3D) phases of the outbreaks. The coverage was 95% under the Cori method when ${p}_{W}=1$; however, lower *p*_*W*_ values led to lower coverage values. In contrast, under the OG1 method, since temporal aggregation of the daily data eliminates the WRE, the coverage was independent of *p*_*W*_ and coverage values close to 95% were obtained.

**Fig. 4:**
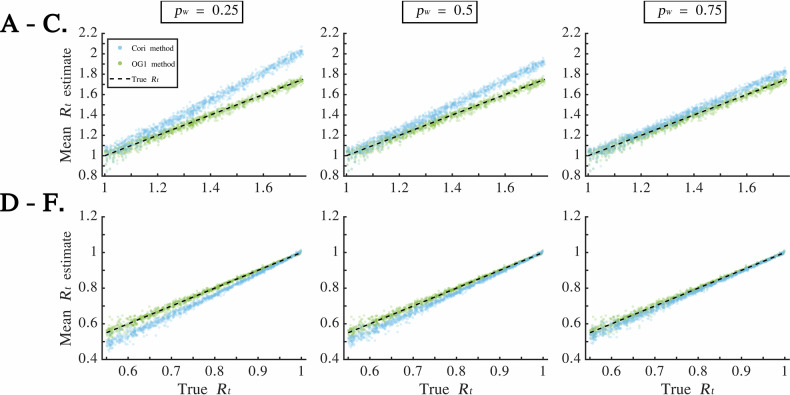
The effect of the true value of *R*_*t*_ on the accuracy of *R*_*t*_ estimates obtained from the Cori and OG1 methods in the presence of WREs. **A**. Scatter plot demonstrating how *R*_*t*_ estimates from the Cori (blue) and OG1 (green) methods in the outbreak growth phase vary for different true values of *R*_*t*_, for datasets with ${p}_{W}=0.25$. In each dataset, the value of *R*_*t*_ was sampled from a uniform $U(1,\,1.75)$ distribution (this analysis uses the second set of simulated datasets described in the “Simulated datasets” subsection of the Methods). Each scatter point represents one dataset (specifically, the value on the y-axis is the mean of the mean *R*_*t*_ estimates during the outbreak growth phase for that dataset; consequently, there are 1,000 blue points and 1,000 green points). **B**. Identical to A, but for ${p}_{W}=0.5$. **C**. Identical to A, but for ${p}_{W}=0.75$. **D**. Identical to A, but for the outbreak decline phase (during which the true value of *R*_*t*_ was sampled from a uniform $U(0.55,\,1)$ distribution; this analysis uses the third set of simulated datasets described in the “Simulated datasets” subsection of the Methods). **E**. Identical to D, but for ${p}_{W}=0.5$. **F**. Identical to D, but for ${p}_{W}=0.75$

In our final analysis of simulated data, we considered the daily disease incidence datasets generated using a range of different *R*_*t*_ values in the growth and decline phases (i.e., the second and third sets of simulated datasets described in the “Simulated datasets” subsection of the Methods). We again found that, in the outbreak growth phase (in which ${R}_{t} > 1$) the Cori method tended to generate overestimates of *R*_*t*_ (Fig. 4A–C; these results were obtained by analysing the second set of simulated datasets described in the “Simulated datasets” subsection of the Methods), whereas in the outbreak decline phase (in which ${R}_{t} < 1$) the Cori method tended to generate underestimates of *R*_*t*_ (Fig. 4D–F; these results were obtained by analysing the third set of simulated datasets described in the “Simulated datasets” subsection of the Methods). Larger errors in the mean *R*_*t*_ estimates from the Cori method occurred for smaller values of *p*_*w*_ and for true *R*_*t*_ values that were not close to one.

### Influenza in San Francisco, 1918

**Fig. 5:**
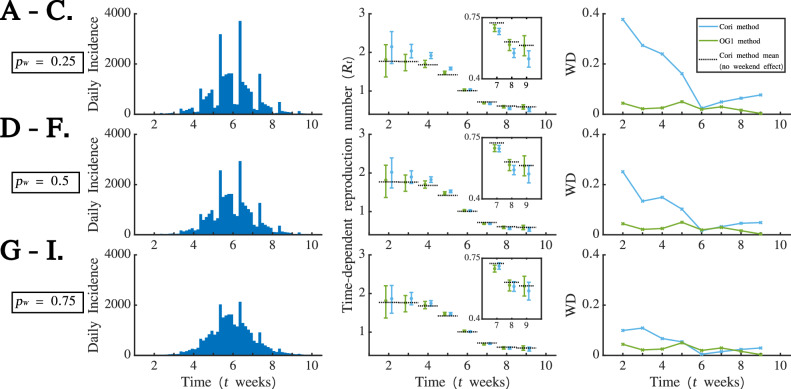
Testing the performance of the Cori and OG1 methods in the presence of WREs for realistic epidemiological data motivated by case reports from San Francisco from the 1918 influenza pandemic. **A.** Daily disease incidence data generated based on the weekly case reports, with a simulated WRE corresponding to ${p}_{W}=0.25$. **B.** Estimates of *R*_*t*_ obtained from the Cori (blue) and OG1 (green) methods for the outbreak shown in panel A; dots represent mean estimates and error bars represent 95% credible intervals. The mean estimate of *R*_*t*_ obtained using the Cori method from the estimated daily data that have not been subjected to a WRE is also shown (black dashed). Insets show zoomed in plots for $t=7,\,8$ and 9 weeks. **C.** The Wasserstein distance between the posterior distribution for *R*_*t*_ obtained using the Cori method from the estimated daily data underlying the dataset in panel A prior to the application of the WRE and either: (i) the posterior distributional estimate of *R*_*t*_ obtained using the Cori method from the dataset shown in panel A (blue); (ii) the posterior distributional estimate of *R*_*t*_ obtained using the OG1 method from the dataset shown in panel A (green). **D**-**F**. Identical to A-C, but for ${p}_{W}=0.5$. **G**-**I**. Identical to A-C, but for ${p}_{W}=0.75.$

We analysed the impacts of WREs on *R*_*t*_ estimates in the context of realistic epidemiological data. To do this, we considered the daily disease incidence time series estimated from the weekly case reports from San Francisco from the 1918 influenza pandemic, subjected to a range of different WREs (see the “Real-world dataset: Influenza in San Francisco, 1918” subsection of the Methods for further details). We considered values of ${p}_{W}=0.25$ (Fig. 5A-C), ${p}_{W}=0.5$ (Fig. 5D-F) and ${p}_{W}=0.75$ (Fig. 5G-I), and estimated *R*_*t*_ using the Cori and OG1 methods.

Unlike in our analyses of simulated data, the true values of *R*_*t*_ underlying the dataset were unknown. Consequently, to assess the accuracy of *R*_*t*_ estimates obtained from the Cori and OG1 methods, we compared them against baseline *R*_*t*_ estimates derived by applying the Cori method to the estimated daily disease incidence before the WRE was applied (baseline mean *R*_*t*_ estimates are shown as black dashed horizontal lines in Fig. 5B, E, H). As in the simulated datasets, we found that mean *R*_*t*_ estimates were more accurate (as assessed by comparing against the baseline mean *R*_*t*_ estimates) using the OG1 method than the Cori method in the presence of WREs. The baseline mean *R*_*t*_ values were more likely to lie within the 95% credible interval under the OG1 method than under the Cori method. We also again found, as expected, that the Cori method performed worst for low values of *p*_*W*_ (Fig. 5B).

To confirm these findings, we calculated the Wasserstein distance (which characterises the distance between probability distributions [29]) between: (i) the posterior *R*_*t*_ estimates obtained from the Cori method applied to the data with WREs and the baseline posterior *R*_*t*_ estimates (i.e. the posterior estimates obtained from the Cori method applied to the data without WREs; blue lines in Fig. 5C, F, I); (ii) the posterior *R*_*t*_ estimates obtained from the OG1 method and the baseline posterior *R*_*t*_ estimates (green lines in Fig. 5C, F, I). Again, this indicated that the OG1 method leads to superior *R*_*t*_ estimates than the Cori method in the presence of WREs, particularly when *p*_*W*_ takes a low value.

## Discussion

Assessing changes in pathogen transmissibility during infectious disease outbreaks is an important challenge [30, 31]. Alongside other measures (e.g., recorded numbers of severe cases or deaths), estimates of quantities such as *R*_*t*_ enable the effectiveness of public health measures to be assessed. Numerous studies have involved estimation of *R*_*t*_ from epidemiological data, for outbreaks of diseases including Ebola [32–36], influenza [37–41] and COVID-19 [42–46].

Here, we have shown that *R*_*t*_ inference using standard methods can be negatively impacted by DOWEs. We considered disease incidence time series that were reported on a daily basis and subject to a WRE, so that some cases occurring at weekends were only reported on the subsequent Monday. Using simulated datasets and daily data derived from weekly case reports from the 1918 influenza pandemic, we estimated *R*_*t*_ using the commonly applied Cori method [1], showing that errors in both the accuracy and coverage of *R*_*t*_ estimates can occur in the presence of a WRE. Larger errors occur when the WRE is strong and *R*_*t*_ is not close to one. In addition to demonstrating the potential negative effects of a WRE on *R*_*t*_ inference, we also presented a method that can be applied to obtain *R*_*t*_ estimates that are both more accurate and generate appropriate coverage values in the presence of WREs. Specifically, we showed that aggregating the daily disease incidence data into weekly values (to remove the WRE) and then using an *R*_*t*_ inference procedure designed for application to weekly data (the OG1 method) can generate robust *R*_*t*_ estimates. Perhaps surprisingly, in the examples that we considered we found that temporal aggregation of the data into weekly values did not negatively impact *R*_*t*_ estimates substantially; even when there was no WRE, the OG1 method generated *R*_*t*_ estimates that were only slightly less accurate than the Cori method and with appropriate coverage values (see Fig. 3).

While differences in estimated *R*_*t*_ values obtained from the Cori and OG1 methods sometimes appeared small when judged by eye, it should be noted that even small errors in *R*_*t*_ estimates can have substantial implications for public health policy. For example, starting from 10 cases with a constant value of ${R}_{t}=1.5$, the expected cumulative number of cases after five generations of transmission is 208. With only a slightly higher value of ${R}_{t}=1.7$, this value becomes 331 cumulative cases. Since projections of case numbers based on inferred *R*_*t*_ values may be used during outbreaks to plan surveillance and control resourcing, such as to determine the contact tracing level required to track the outbreak or the treatment and intensive care unit bed requirements, accurate *R*_*t*_ estimation is of paramount importance.

The research in this article builds on past studies involving the development of *R*_*t*_ estimation methods accounting for delayed case reporting [47, 48]. For example, Bajaj *et al*. [48] developed an inference framework for estimating the reporting delay distribution and *R*_*t*_ simultaneously. When that method is applied, the reporting delay distribution is inferred by considering updates to the disease incidence time series data over time by public health authorities. While that approach accounts for reporting delays when estimating *R*_*t*_, a key difference in the present study is that, rather than a single reporting delay distribution applying to all cases, we considered a reporting delay based on the day of the week (specifically, a WRE under which delayed reporting was specifically assumed to occur over weekends).

Although applications of most methods for estimating *R*_*t*_ have not considered DOWEs, there are some exceptions [43, 49]. For example, in the widely used EpiNow2 software package [50], there is an option for the user to specify a probability distribution relating true case numbers to observations on different days of the week. However, this requires such a reporting distribution to be known in advance, and robust parameterisation would be contingent on the availability of reliable reporting propensity data. Pang *et al*. [51] presented a method using generalised additive models (GAMs) to derive growth rates by smoothing surveillance data through the parametric incorporation of DOWEs. The authors then made structural assumptions about the mechanisms underlying transmission to translate the inferred growth rates into *R*_*t*_ estimates, and compared their inferred *R*_*t*_ values with estimates obtained from the Cori method. However, GAMs require sufficient data to ascertain a valid fit, meaning caution is needed with the application of such an approach to small outbreaks.

As with any study, our analyses involved assumptions. For example, we assumed that delayed reporting at weekends led to some cases being reported on the subsequent Mondays, but not other subsequent days. In reality, WREs may be more complex; for example, cases affected by delayed weekend reporting could be distributed throughout the subsequent week, rather than all being reported immediately after each weekend on the following Monday. We note that, in principle, the OG1 method could still be applied in such a scenario by choosing to aggregate cases into weekly values using a Saturday-Friday window; by making that choice, all cases that were subject to delayed reporting over weekends and were reported at any time in the subsequent week would be included in the correct week in the weekly aggregated data, without requiring detailed knowledge about the WRE.

Another limitation of our analyses is that we did not attempt to account for WREs when we applied the Cori method. While this is a realistic representation of how standard *R*_*t*_ inference methods are typically applied in practice, in principle it is possible to estimate *R*_*t*_ from daily data without aggregating the data but while still accounting for WREs. One possibility is to build the WRE mechanistically into the *R*_*t*_ inference procedure, however this may require the WRE to be characterised accurately (both in terms of the extent of the WRE and precisely when unreported cases at weekends are ultimately reported). As noted above, an important benefit of our approach involving applying the OG1 method to weekly aggregated data is that it is not necessary to know precisely how the WRE is affecting the daily reported data. For example, the OG1 method as applied here provides the same *R*_*t*_ estimates irrespective of the value of *p*_*W*_, and (subject to an appropriate choice of weekly aggregation window) would provide the same estimates irrespective of whether the WRE leads to weekend cases being reported on Mondays or any other days within the subsequent week. An alternative way in which DOWEs could be accounted for under the Cori method framework might be to smooth the daily incidence data to reduce the DOWE before applying the Cori method. A preliminary analysis utilising this approach in the context of WREs is presented in Supplementary material 1: Fig. S2, demonstrating that smoothed disease incidence data can bring *R*_*t*_ estimates from the Cori method more in line with those from the OG1 method. However, we contend that rather than being preferable, such an approach is simply an alternative to applying the OG1 method (indeed, in Supplementary material 1: Fig. S2, slightly more accurate estimates are obtained from the OG1 method than from the Cori method applied to smoothed disease incidence data). Furthermore, a modelling choice is required regarding the precise method to use to smooth the daily disease incidence data, increasing the chance that model-based assumptions bias the resulting *R*_*t*_ estimates.

We applied the OG1 method in the context of weekly aggregated data. However, another approach for inferring *R*_*t*_ from weekly aggregated disease incidence time series data was developed by Nash *et al*. [52]. That method could also be used following data aggregation in future analyses to reduce the impacts of WREs on *R*_*t*_ estimates, in a similar fashion to application of the OG1 method here. We considered simulated and real-world data for influenza, for which the serial interval is relatively short (specifically, the serial interval distribution that we used had a mean value of 2.6 days [25]). For other pathogens with longer serial intervals, for which realised serial intervals may be distributed over a larger number of days, the impact of WREs on *R*_*t*_ estimates may be reduced (for an initial analysis indicating this, see Supplementary material 1: Fig. S3, however a more in-depth investigation is required to confirm the generality of this result). Finally, in our analyses we assumed that cases occurring on weekdays were reported immediately. In addition to a WRE, we could have instead assumed that cases reported on weekdays were subject to delayed reporting. However, our goal was to analyse the impact of WREs on *R*_*t*_ inference; additionally including delayed weekday reporting would have conflated errors due to weekday reporting delays and the WRE.

## Conclusions

Despite the simplifications underlying our analyses, we were able to demonstrate the key concept that WREs can beset *R*_*t*_ inference. We also showed that the OG1 method [23], if applied following weekly aggregation of daily disease incidence time series data, can be used to obtain robust *R*_*t*_ estimates in the presence of WREs.

The reliability of *R*_*t*_ estimates in the presence of reporting artefacts has practical implications for public health policy making. Since inferred *R*_*t*_ values are among the metrics used to guide public health measures during outbreaks, robust estimates can promote evidence-based planning and efficient allocation of surveillance and control resources. This includes more reliable estimation of required healthcare capacity, hospital staffing, contact tracing personnel and testing infrastructure. Improved *R*_*t*_ estimation may help to avoid unnecessary public health or economic costs associated with interventions that are implemented or maintained based on biased *R*_*t*_ estimates. Furthermore, by providing more reliable situational awareness during an outbreak, accurate *R*_*t*_ inference not only improves operational decision making but also enhances policy makers’ ability to communicate the risk posed by a pathogen to the public appropriately.

Since WREs, and DOWEs more generally, are commonplace in epidemiological data, application of our proposed approach could enable more robust *R*_*t*_ estimates to be generated. We hope that this will promote effective public health decision making during future infectious disease outbreaks.

## Supplementary information

Supplementary material 1: Supplementary text, Supplementary table (Table S1) and Supplementary figures (Figs S1-S3)

## Data Availability

The analyses presented in this manuscript were conducted using code written in MATLAB (version R2021b; developed by The MathWorks, Inc.). This computing code is available, along with relevant data, in our public GitHub repository [53].
