# Supplementary material for: Robust estimation of the time-dependent reproduction number in the presence of weekend reporting effects
Source: BMC Glob Public Health. 2026 May 21;4:48. doi: 10.1186/s44263-026-00280-z (PMC13195961; doi:10.1186/s44263-026-00280-z)
Supplement: Supplementary file 1 — Supplementary material 1: Supplementary text, Supplementary table (Table S1) and Supplementary figures (Figs S1-S3) [file 44263_2026_280_MOESM1_ESM.docx]

**Supplementary material 1**

**Supplementary Material for *Robust estimation of the time-dependent reproduction number in the presence of weekend reporting effects***

I Ogi-Gittins, N Steyn, AR Kaye, EM Hill, RN Thompson

Supplementary text

Here, we describe how we inferred the daily numbers of pandemic influenza cases in San Francisco from 23 September to 24 November 1918 from weekly case reports [1], following the method described by Sachak-Patwa *et al.* [2].

We considered the Susceptible-Exposed-Infectious-Removed (SEIR) model, given by

$$\frac{dS}{dt}=-\frac{\beta SI}{N_{e}}, \frac{dE}{dt}=\frac{\beta SI}{N_{e}}-\kappa E, \frac{dI}{dt}=\kappa E-\mu I, \frac{dR}{dt}=\mu I,$$

in which $S(t)$, $E(t)$, $I(t)$ and $R(t)$ are the numbers of susceptible, exposed, infectious and removed individuals at time $t$ (which is measured in weeks here). Under this model, at any time we assume that $S\left( t \right)+E\left( t \right)+I\left( t \right)+R\left( t \right)=N_{e}$, where $N_{e}$ is the effective population size. The value of $N_{e}$ is less than the total population size of San Francisco; this modelling assumption reflects the fact that pathogens are most likely to be transmitted within individuals’ contact networks (so not all susceptible individuals are available for infection).

Under this model, the number of newly reported cases in the one-week period $[t-1,t]$ is $\int_{t-1}^{t} \kappa E\left( j \right)dj$, which can be approximated by $\kappa E\left( t \right)$. Hence, the number of cases in week $t$ is assumed to be $\kappa E\left( t \right)$.

We approximated the initial conditions $E\left( 0 \right)$, $I\left( 0 \right)$ and $R\left( 0 \right)$ from the disease incidence dataset using the approach described in section S5 of the Supplementary Material of the article by Sachak-Patwa *et al.* [2]. Then, $S\left( 0 \right)=N_{e}-E\left( 0 \right)-I\left( 0 \right)-R(0)$.

We set $1/\kappa=4/7$ weeks and $1/\mu=1$ week [3], and then estimated the values of $\beta$ and $N_{e}$ by fitting the SEIR model to data from the weekly case reports using least squares estimation (where the model output was generated by numerically solving the SEIR model using the Matlab function ode45). The resulting fit is shown in Fig S1, and the model parameter values are given in Table S1.

To recover the daily number of cases in the absence of a WRE, we then converted the estimated parameter values from weekly values to their corresponding daily values (i.e., we set $\beta=0.729$ per day, $1/\kappa=4$ days and $1/\mu=7$ days), numerically solved the SEIR model again using ode45 (but now with $t$ measured in days) and recorded $E\left( t \right)$ each day. We then set $I_{t}^{\mathrm{daily}}=\kappa E\left( t \right)$ for $t=1,2,3\ldots$.

Supplementary table

**Table S1: Parameter values (and estimated initial conditions) in the SEIR model after fitting to the weekly incidence data from San Francisco from the 1918 influenza pandemic using least squares estimation.**

| **Parameter** | **Description** | **Value** | **Source** |
| --- | --- | --- | --- |
| $\beta$ | Infection rate | 5.1 week^-1^ | Estimated |
| $N_{e}$ | Effective population size | 29,601 | Estimated |
| $1/\kappa$ | Latent period | 4/7 weeks | [3] |
| $1/\mu$ | Infectious period | 1 week | [3] |
| $(S\left( 0 \right),E\left( 0 \right),I\left( 0 \right), R(0))$ | Initial number of individuals in each compartment | (29570, 25, 6, 0) | Estimated using method in [2] |

Supplementary figures

**Figure S1: Comparison of the output of the SEIR model (specifically, the number of newly reported cases each week) with parameters and initial conditions given in Table S1 (blue bars) and the weekly incidence data from San Francisco from the 1918 influenza pandemic (red dots).**

**Figure S2: Analogous results to Fig 2 in the main text, but with the disease incidence data smoothed before the Cori method was applied.** In the analyses shown here, the disease incidence data in panel A were smoothed before the Cori method was applied to generate the results shown in red in panels B and C. In particular, boxcar smoothing was used, with the disease incidence on any given day $i$, $I_{i}^{daily}$, replaced by the mean value across seven days centred on day $i$, ${{\{I}_{s}^{daily}\}}_{s=i-3}^{i+3}$. For the first three and last three days in each dataset, days before and after the dataset were omitted from the calculation (so that, for example, $I_{1}^{daily}$ was replaced with the mean of ${{\{I}_{s}^{daily}\}}_{s=1}^{4}$). For each week $t$, the incidence data within that week were then multiplied by a constant factor chosen to ensure that the smoothed daily incidence across week $t$ sum to the same number of cases as in the original (unsmoothed) daily disease incidence dataset. Similar smoothing was performed on the data in panels D and G to generate the results shown in red in panels E-F and H-I, respectively.

**Figure S3: Initial investigation into the combined effects of different** $\boldsymbol{R}_{\boldsymbol{t}}$ **values and serial intervals on** $\boldsymbol{R}_{\boldsymbol{t}}$ **estimates obtained when the Cori method is applied, for** $\boldsymbol{p}_{\boldsymbol{w}}\boldsymbol{=0.5}$**.** To generate this figure, for a given mean serial interval value (shown on the y-axis), the shape parameter of the (gamma distributed) serial interval distribution was set to four (as in the serial interval distribution used in our other analyses) and the rate parameter was chosen to give the specified mean. For each ($R_{t}$, mean serial interval) pair, a simulation was generated using a deterministic renewal equation (i.e., using equation (1) in our manuscript without sampling from a Poisson distribution) starting from $I_{1}^{daily}=100$ (on a Monday) and assuming that $R_{t}$ takes a constant value throughout the simulation. The simulation was run either for six weeks or until the end of the first week in which the daily incidence exceeded 1,000 cases (whichever was sooner). Then, a deterministic WRE corresponding to $p_{W}=0.5$ was applied. The value of $R_{t}$ was then estimated each week using the Cori method, and the mean estimate was compared against the true value of $R_{t}$ used to generate the dataset. The percentage error in the $R_{t}$ estimate was then calculated and averaged across the simulated weeks (from $t=2$ weeks onwards). This preliminary analysis indicates that, for long and highly dispersed serial interval distributions, the error in $R_{t}$ estimates due to WREs may be less than for short and less dispersed serial interval distributions.

References

1. Chowell G, Nishiura H, Bettencourt LMA. Comparative estimation of the reproduction number for pandemic influenza from daily case notification data. J R Soc Interface. 2007;4: 155–166.

2. Sachak-Patwa R, Byrne HM, Thompson RN. Accounting for cross-immunity can improve forecast accuracy during influenza epidemics. Epidemics. 2020;34: 100432.

3. Tuite AR, Greer AL, Whelan M, Winter A-L, Lee B, Yan P, et al. Estimated epidemiologic parameters and morbidity associated with pandemic H1N1 influenza. Can Med Assoc J. 2010;182: 131–136.
